# Experimental Characterization of RGB LED Transceiver in Low-Complexity LED-to-LED Link

**DOI:** 10.3390/s20205754

**Published:** 2020-10-10

**Authors:** Mariam Galal, Wai Pang Ng, Richard Binns, Ahmed Abd El Aziz

**Affiliations:** 1 Faculty of Engineering and Environment, Northumbria University, Newcastle upon Tyne NE1 8ST, UK; Mariam.abdelmoteleb@northumbria.ac.uk (M.G.); Richard.binns@northumbria.ac.uk (R.B.); 2 College of Engineering and Technology, Arab Academy for Science, Technology & Maritime Transport, Alexandria P.O. 1029, Egypt; ahmed.abdelaziz@aast.edu

**Keywords:** LED photodetector, LED-to-LED communication, BER, matched filter, visible light communication (VLC)

## Abstract

This paper proposes a low-complexity and energy-efficient light emitting diode (LED)-to-LED communication system for Internet of Things (IoT) devices with data rates up to 200 kbps over an error-free transmission distance up to 7 cm. The system is based on off-the-shelf red-green-blue (RGB) LEDs, of which the red sub-LED is employed as photodetector in photovoltaic mode while the green sub-LED is the transmitter. The LED photodetector is characterized in the terms of its noise characteristics and its response to the light intensity. The system performance is then analysed in terms of bandwidth, bit error rate (BER) and the signal to noise ratio (SNR). A matched filter is proposed, which optimises the performance and increases the error-free distance.

## 1. Introduction

The Internet of Things (IoT) requires that more and more devices be connected to each other. These devices are not just limited to computers and smartphones, but also include common household appliances such as washing machines, coffee machines, refrigerators as well as personal clothes and toys [1]. It is common nowadays to have tens or even hundreds of devices connected to the internet in one household. The connectivity for those smart appliances and devices is putting a great pressure on the spectrum of the radio frequency (RF) communication, which is already overcrowded [2]. Many researchers are therefore focused on finding out-of-the-box solutions for this so-called ‘spectrum crunch’ by proposing smaller cell-sizes, where frequency reuse can be employed. In these nano and femto-cells, the short-range and low-bandwidth communications between IoT devices can be achieved without overstressing the RF spectrum [3]. It remains a challenge however to find the appropriate low-power, low-complexity and low-cost communication technology to serve the needs for IoT devices. wireless fidelity (Wi-Fi) and Bluetooth low energy (BLE) are the “go-to” option for many of those smaller devices. While Wi-Fi operates on relatively high power and has an extended range, BLE is favoured as standard in many IoT applications because of its relatively high data rate (up to 2 Mbps) and its range extending to 200 m [4]. However, both Wi-Fi and Bluetooth are prone to eavesdropping and can cause interference with other RF technologies [5]. They are therefore not used in RF sensitive environments such as in some hospitals and control rooms. Similarly, many users refuse to rely on them for transmission of secure data such as during contactless payment or personal identification because they are easy to hack. These drawbacks provided a rich ground for the use of visible light communication (VLC) in IoT devices especially in indoor environments [6].

As a complementary technology to RF, VLC is gaining popularity due to its low energy consumption and inherent security against eavesdropping [6]. VLC signals cannot penetrate walls and obstacles and are therefore confined within a room, safe from hacking by outsiders not in line of sight with the transmitter. Light emitting diodes (LEDs) are at the core of contemporary energy efficient lighting, which at the same time, can be deployed as VLC transceivers, a poignant step towards green communications. Due to its low power consumption, its small cell size and its immunity to interference from RF, VLC is considered a major player in the era of 5G and IoT [7].

The research in visible light communication is currently divided into two paths: on the one hand, researchers are pushing the bandwidth, range, and speed limits of VLC to compete with and complement high-end RF communication technologies. On this path, VLC is used for high-speed data streaming, internet connection and telephony. For instance, the company PureLifi provides a VLC solution to equip homes and offices with high-speed internet and connectivity over visible light [8]. Researchers from Fraunhofer Heinrich Hertz Institute proposed a 500 Mbps bidirectional VLC system using orthogonal frequency division multiplexing (OFDM) as modulation technique [9]. A 5 m free-space link based on a blue laser diode is proposed in [10], which can provide up to 9-Gbps quadrature amplitude modulation (QAM)-OFDM communication over visible light. Those high-speed solutions mark VLC as a high-speed communication technology and enable it to compete with other RF technologies in this rank. However, they are expensive and complex and are therefore considered an overkill for simple IoT devices. On the other hand, the other path considers employing VLC in applications that require low cost of implementation, wide availability, and easy commercial deployment. These applications need only infrequent data transmission, low data throughput and low communication speeds over a short range [11]. The system proposed in this paper follows that low-cost-of-implementation path.

A traditional VLC system transmitter consists of an LED controlled by a driver circuit to transmit modulated light at a higher flickering rate than the human eye can perceive. The most commonly used photodetector circuit is usually equipped with a PIN or avalanche photodiode followed by a transimpedance amplifier (TIA) [7]. Such VLC system configuration however has the limitation of having a single directional, simplex channel nature. If a bidirectional or duplex system is needed, another transmitter-receiver pair must be implemented, which doubles the cost, space and complexity of the system. This sets VLC back in comparison to other RF technologies such as Bluetooth or Wi-Fi. Those latter technologies possess a single antenna on each end of the communication system acting as both a transmitter and receiver for RF signals.

LED-to-LED communication is a subcategory of VLC that employs the same LED for transmitting and receiving signals, decreasing thereby the cost, complexity, energy and space requirements even further. It reduces the cost of the system by replacing the costlier photodetector and upgrades the simplex channel of the VLC system to time-multiplexed half-duplex channel, hence halving the total number of transceiver devices [12].

The structure of a PN junction allows the LED to sense an incoming beam of light with zero or reverse bias [13]. Although its sensitivity is low compared to the PIN photodiode (due to the missing intrinsic layer), it can be employed in simple low-data-rate, short-distance and low-cost IoT devices [14,15]. Dietz et al., demonstrated the potential of using the LED as photosensor for ambient light by making use of its junction capacitor [12]. Employing such method, Disney Research Lab, Zurich, Switzerland, achieved a toy-to-toy communication channel at 1 kbps over small distances reaching 5 cm [8,9]. Giustiniano et al., proposed a similar system with data rates up to 14 kbps, reaching a distance of 20 cm based on the same principle of operation [16]. However, depending on the internal capacitor of the LED for photodetection is not a reliable solution. The capacitance varies according to the make and model of the LED. Furthermore, the long discharging time results in slow communication.

Galal et al., showed the LED’s ability as photodetector where the sub-LEDs of an RGB LED can have a bandwidth up to 6 MHz when reverse biased [13]. The same study also concluded that such LED could detect light in photovoltaic mode (with no bias) and when slightly forward biased up to 2V. Furthermore, the experimental results proved that the red LED has the highest responsivity out of all three sub-LEDs to the light emitted from the green sub-LED. Hence the combination of red-to-green has been identified as the best-case scenario for a successful LED-to-LED link and is therefore adopted in this paper. A 100 Mbps LED-to-LED communication system was designed by Stepniak et al., [17] and Chun et al., [18]. The high data rate was achieved through design optimization of a receiver circuit and the careful choice of high-quality communication LEDs along with complex modulation and wavelength division multiplexing techniques. The proposed systems in [17] and [18] are based on expensive and specifically designed single-colour LEDs, as well as complicated optics and heavy offline processing. Such a solution is too complex for IoT devices. Many researchers have already proposed VLC systems with RGB LEDs as transmitters and light sources, but not as photodetectors. For instance, Shrestha et al., proposed an indoor VLC system employing an array of RGB LEDs as transmitters on the ceiling, which has a range of 2 m and can provide data rates up to 19.2 kbps [19].

To the best of the authors’ knowledge, the gap in the field of LED-to-LED communication mentioned in literature is threefold: Firstly, only single-coloured (mostly red and amber) LEDs are employed. Secondly, the research focuses on increasing the data rate and system performance, increasing thereby the cost and complexity by using complex lenses and concentrators [18]. This disagrees with the nature of communication required by typical IoT devices. Finally, the energy response and noise characteristics of an off-the-shelf RGB LED as a photodetector have not been investigated.

In order to fill those gaps, this paper proposes an LED-to-LED system based on off-the-shelf RGB white LEDs which are more favourable for lighting and indicators in IoT than single-coloured LEDs. It also offers reduced cost and complexity by stripping the system of all complicated circuitry, optics and processing-hungry techniques. This paper implements and experimentally verifies the proposed system with off-the-shelf components costing as low as USD 2.60 per communicating device. Finally, this paper studies the noise characteristics of the LED photodetector and verifies that it is additive white Gaussian noise (AWGN). For that reason, a matched filter is proposed to achieve error-free transmission over a 7 cm distance with a speed up to 200 kbps, reaching thereby better performance compared to a PIN photodiode employed in the same zero bias scenario and meeting the requirements for IoT devices.

The proposed low-complexity LED-to-LED link can be applied to any application that requires low data rate and short-range line-of-sight communication. It is especially useful in applications where small chunks of secure data are transmitted, such as the exchange of biometric information, user identification, contactless payment as well as for communication between smart devices in a personal or body area network or for infotainment systems [20]. Other applications that could benefit from the LED-to-LED communication include sensing applications such as high-accuracy distance sensors (as replacement for ultrasonic distance sensors) and biomedical sensors [12]. Last but not least, LED-to-LED has a high potential for being used in bidirectional opto-coupling and opto-insulating applications.

## 2. Experimental Setup

White light can be produced using either blue-phosphorus or RGB LEDs. While the former is not suitable for LED-to-LED communication because the phosphorus coating prevents the LED from receiving light, the latter can emit and detect simultaneously through three independent channels [13]. This paper utilizes common cathode RGB LEDs (Kingbright) on both ends of the communication link. The green and red sub-LEDs are employed for transmission and photodetection respectively, as recommended by the findings in [13].

**Figure 1:**
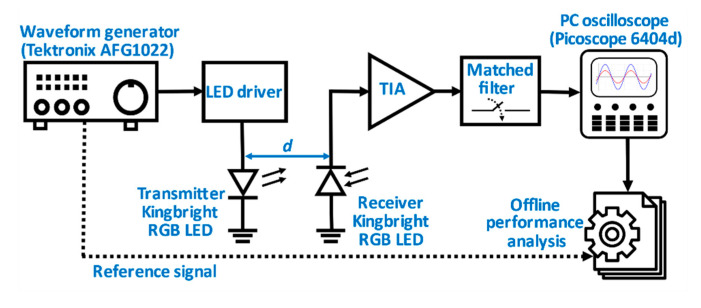
Block diagram of the proposed experimental setup.

The block diagram of the experimental setup is shown in Figure 1. The transistor-based LED driver biases the green sub-LED with 15 mA while keeping the energy consumption at a minimum and allows fast on-off switching of the transmitter LED [21]. The waveform generator transmits a non-return-to-zero (NRZ) on-off-keying (OOK) modulated cyclic and pseudorandom binary sequence (PRBS) of length 2^20^ − 1 bits at 10, 50, 100 and 200 kbps. The sequence is generated using linear feedback shift registers applying the monic polynomial:

(1) $$PRBS20=x^{20}+x^{3}+1$$

For testing the LED’s response and noise characteristics as photodetector, the waveform generator is replaced with a constant (DC) signal to keep the transmitter LED on.

**Figure 2:**
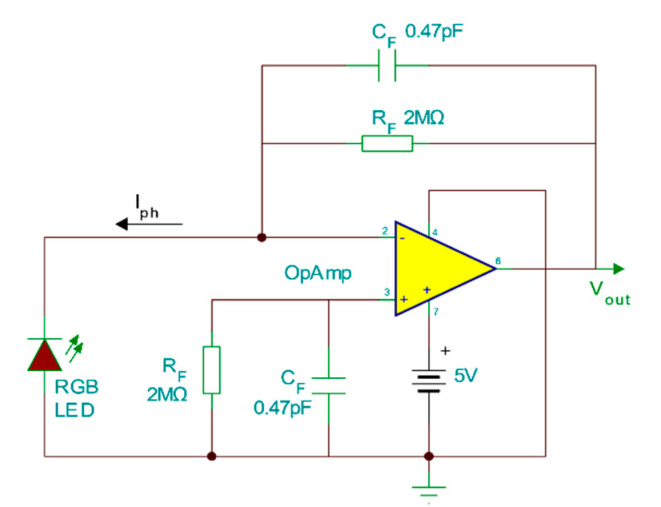
Transimpedance amplifier (TIA) circuit diagram.

At the receiver end with a varying channel distance d an identical RGB LED (to the transmitter) is connected to the TIA based on an operational amplifier chip (OPA380) as seen in Figure 2. The transmitter and receiver circuits are galvanically completely separated. For comparison purposes, the receiving LED is replaced with a PIN photodiode (BPW34 Vishay Semiconductors) under the same measurement conditions. Since reverse biasing the LED has only a negligible effect on improving its bandwidth and sensitivity [13] unlike PIN photodiodes, the photovoltaic mode is chosen for the detection circuit. The detected signal from the TIA feeds into an optimised matched filter, to mitigate the LED photodetector noise effect and is captured on an oscilloscope (Picoscope 6404D). Offline processing is for performance analysis.

To achieve good synchronization between the transmitter and receiver circuits and hence a minimum BER, a preamble consisting of a sequence of ‘1’ and ‘0’ bits is transmitted at the begin of the transmission phase. The detecting circuit uses this preamble to detect the rising edge, know the exact bit duration and adjust the threshold. After the preamble, the detecting processor can sample at a time that roughly falls at the middle of the received bit (at the widest eye opening).

The proposed system employs only a single 5 V power supply and consumes a maximum of 110 mW. Care has been taken to choose components which are cheap and available off-the-shelf. The low price and power consumption are attractive features for IoT device manufacturers and mass producers. The experiments take place in a well-lit lab with overhead artificial lighting placed at a distance of approximately 2 m from the testing bench. Since the ambient light is perpendicular to the transmission direction of the LED-to-LED link and the cone of acceptance of the LED is proven to be only 18° according to [12], the ambient light has no effect on the receiver LED. Furthermore, the LED photodetector sensitivity is too low to detect indirect ambient light from the ceiling lights [13,22]. For those reasons, the ambient light is found to have a negligible effect on the experimental results presented in this paper.

## 3. Results and Analysis

The compiled results are the averages of the series of repeated measurements in all experiments.

### 3.1. Response of RGB LED Photodetector

**Figure 3:**
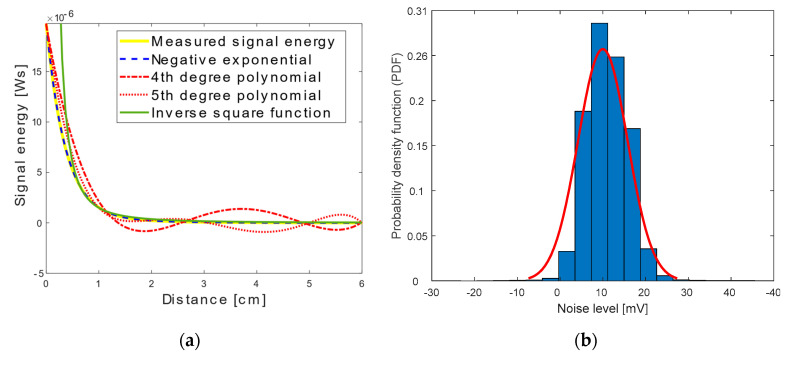
Characteristics of a light emitting diode (LED) photodetector: (**a**) received signal energy decay with increasing distance; (**b**) histogram of LED photodetector noise signal and its fitting to a Gaussian function.

The aim of this experiment is to deduce the response of the RGB LED as photodetector to incoming light at different energy levels and to relate the signal decay response to a well-known function. The obtained energy levels at each distance are shown in Figure 3a. The graph shows a significant but expected drop up to a transmission distance of 1 cm and proves that the LED photodetector can be described by the inverse square law of light $1/r^{2}$ at distances longer than 0.5 cm [10]. The measured signal energy also shows an overlap with the negative exponential function. Therefore, the response can be best described as a two-term exponential function as follows:

(2) $$E=1.9\times 10^{-5}\times e^{-2.8d}+9.1\times 10^{-7}\times e^{-0.89d}$$

where d is the transmission distance.

### 3.2. LED Photodetector Noise Measurement

In order to analyse the noise of the LED as a photodetector, a DC signal is transmitted. Then, the DC component of the received signal is subtracted to retrieve the receiver noise. Figure 3b depicts the resultant noise probability density function has a normal distribution, as expected. The performed FFT indicates that the noise is white. Hence, the noise of the LED photodetector is defined to be additive white Gaussian noise (AWGN).

### 3.3. Bandwidth of the LED Receiver Circuit

**Figure 4:**
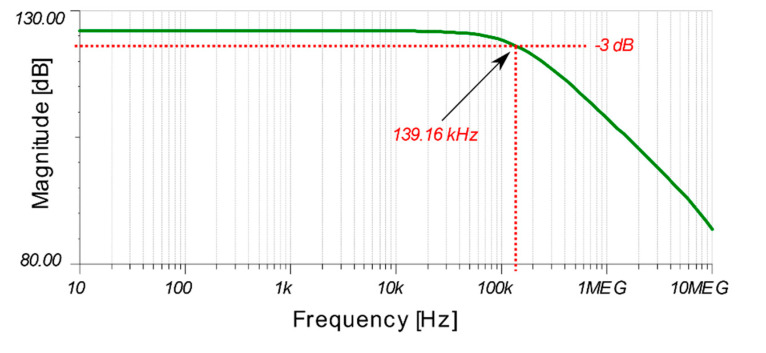
Bode plot of TIA receiver circuit.

The bandwidth of the LED receiver circuit with the proposed TIA is practically investigated to identify the maximum data rate and its simulated Bode plot is shown in Figure 4. The Bode plot suggests that the TIA circuit acts as a low pass filter with a 3 dB bandwidth of around 140 kHz. For a non-return-to-zero (NRZ) OOK signal with one level per bit, this bandwidth allows a maximum bit rate of 280 kbps. To verify this result practically, the rise time $t_{r}$ of the received signal is measured (calculated between 10% and 90% on the rising voltage edge) [23]. $t_{r}$ is found to be 3.8 µs, which translates to a bandwidth $BW_{3dB}$ of 90 kHz according to the equation [23]:

(3) $$BW_{3dB}\;=\;\frac{0.35}{t_{r}}$$

This 90 kHz bandwidth allows OOK data transmission at speeds up to 180 kbps. For that reason, data rates of only up to 200 kbps are investigated. At higher frequencies, the TIA fails to saturate to the upper voltage rail, hence acting as a low pass filter/integrator and turning the received square wave close to a triangular shaped waveform.

### 3.4. BER Improvement with Matched Filter

Now that the photodetector noise has been determined, the bit error rate (BER) and the SNR of the system can be deduced to investigate the performance of the system. The BER can be measured using two methods:

The error bits are deduced from the generated PRBS20 bits to calculate the BER:

(4) $$BER\;=\;\frac{error\;bits}{total\;number\;of\;bits}$$

**Figure 5:**
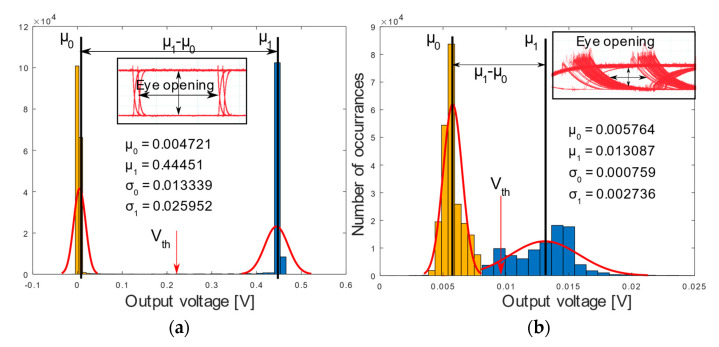
Eye diagram (inset) and signal constellation in (**a**) back-to-back configuration and (**b**) at 10 cm distance.

In order to verify the measured BER results, the BER is also calculated via the quality factor (Q-factor) obtained from the captured eye diagram [24]:

(5) $$Q\;=\;\frac{\left|\mu_{1}-\mu_{0}\right|}{\sigma_{1}+\sigma_{0}}$$

where $\mu_{1}$ and $\mu_{0}$ are the mean voltages for the ‘1’-bit voltage rail and ‘0’-bit voltage rail and $\sigma_{1}$ and $\sigma_{0}$ are the standard deviation values of the ‘1’ and ‘0’ rails due to the AWGN respectively. Hence, the BER can be calculated using the following relation [24]:

(6) $$BER\left(V_{th}\right)=\frac{1}{2}\left[erfc\left(\frac{\left|\mu_{1}-V_{th}\right|}{\sigma_{1}}\right)\;+\;erfc\left(\frac{\left|V_{th}\;-\;\mu_{0}\right|}{\sigma_{0}}\right)\right]$$

where $V_{th}$ is the threshold voltage which determines where the decision is made. In this scenario it is taken as the centre rail between the ‘0’ and ‘1’. The histograms for 0 and 10 cm at 10 kbps as well as the eye diagrams and Q-factor’s parameters are depicted in Figure 5a,b, respectively. For the back-to-back case in Figure 5a, the two rails of the signal are distinct and far away from each other, allowing a right decision to be taken with $V_{th}$ at the center. This agrees with the wide eye opening. On the contrary, this distinction vanishes in the signal constellation at 10 cm distance as seen in Figure 5b, where the normal distribution functions of the ‘0’ and ‘1’ rails are overlapping. In agreement therewith, the eye is closed, which corresponds to a higher probability of error.

**Figure 6:**
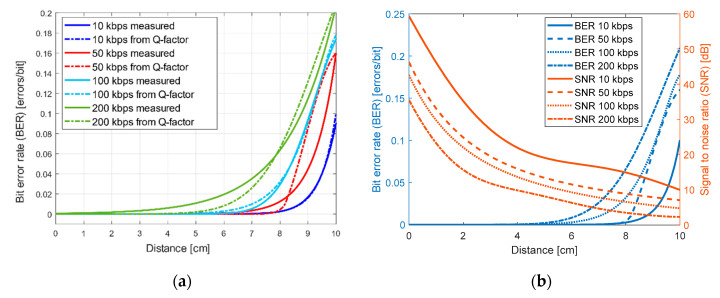
(**a**) Comparison between the measured and calculated BER; (**b**) BER and SNR at different distances and data rates.

The comparison between the measured BER results employing Equation (4) and the calculated BER from Equation (6) for all data rates shows less than 9% deviation between the two methods as seen in Figure 6a. The deviation is calculated by subtracting the measured from the calculated BER and dividing by the measured (true) value. Hence, both approaches have similar responses which validates the practical approach.

Figure 6b shows the average BER at varying distances. As expected, the BER rises with longer transmission distance and higher data rate. Error-free data transmission can be achieved at a maximum distance of 1 cm at 200 kbps, 1.5 cm at 100 kbps and 2 cm at 50 kbps while it reaches 3 cm at 10 kbps. The BER stays below the acceptable 10^−3^ range [17] up to 7 cm for 10 and 50 kbps, up to 4 cm for 100 kbps and up to 3 cm for 200 kbps.

**Figure 7:**
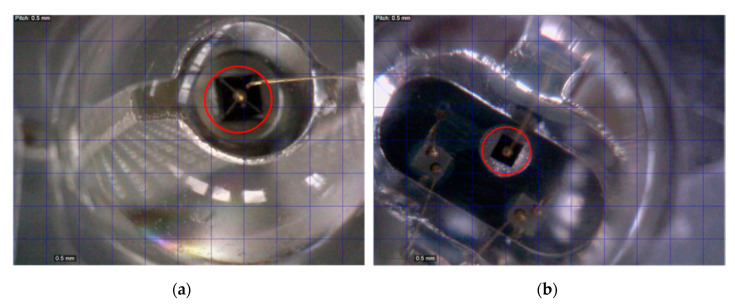
Images of the active areas of (**a**) single-colour (red) LED and (**b**) red sub-LED of RGB LED when seen under the microscope at the same scale.

This low sensitivity is because an RGB LED has a much smaller active area for light emission and detection per sub-LED compared to a regular single-colour LED. This is apparent after placing both LEDs under a microscope and comparing their active areas when dimly lit. The active area of the single-colour LED is 1.99 times larger than that of the sub-LED of the RGB LED, which means only 50% of light is captured compared to single-colour LED. Figure 7 compares the active areas (circled in red) of the single-colour red LED in (a) with that of the red sub-LED in a typical off-the-shelf RGB LED in (b) under the same scale. Moreover, it can be concluded that the small data rates achieved by this small-area LED photodetector can be interpolated to match the higher data rates achieved in Stepniak’s research [17].

The signal-to-noise ratio (SNR) can be determined from the BER of the NRZ OOK modulation as follows [23]:

(7) $$BER\;=\;\frac{1}{2}erfc\left(\frac{1}{2\sqrt{2}}\sqrt{SNR}\right)$$

The graph in Figure 6b also compares the SNR for the LED-to-LED communication link at different distances for data rates of 10, 50, 100 and 200 kbps. The maximum SNR achieved is 50 dB at the back-to-back configuration when transmitting at 10 kbps. It drops to 35 dB when the speed increases to 200 kbps. As expected, the SNR decays exponentially along the distance and at higher speeds. In the worst-case scenario, the SNR reaches 2.3 dB when transmitting at 200 kbps over a distance of 10 cm.

The matched filter technique is a special type of maximum likelihood receivers which is known to maximize the signal to noise ratio of a received signal suffering from AWGN [7], such as in the RGB LED photodetector.

**Figure 8:**
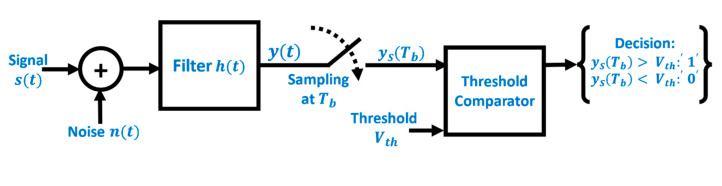
Block diagram of a digital communication system.

The block diagram in Figure 8 demonstrates a typical digital communication system. In order to minimize the BER of the system, the SNR needs to be maximized by a filter at the comparator stage. At the input point of the threshold comparator block, the AWGN power $N_{p}$ can be derived in terms of the its power spectral density PSD, which is equal to $N_{0}/2$ as follows [23]:

(8) $$N_{p}=\overset{\infty }{\underset{-\infty }{\int }}PSD\;df\;=\;\frac{N_{0}}{2}\;\overset{\infty }{\underset{-\infty }{\int }}{\left|H\left(f\right)\right|}^{2}\;df$$

where H(f) is the transfer function of the filter.

Similarly, the following Equation displays the power of the signal $S_{p}$ at the input of the threshold comparator block:

(9) $$S_{p}={\left|y_{s}\left(T_{b}\right)\right|}^{2}=\;{\left|\int_{-\infty }^{\infty }S\left(f\right)H\left(f\right)e^{-j2\pi fT_{b}}\right|}^{2}$$

where $y_{S}\left(T_{b}\right)$ is the sampled filter output signal at $T_{b}$.

Dividing the signal power by the noise power to get the signal to noise ratio SNR:

(10) $$SNR=\;\frac{S_{p}}{N_{p}}=\frac{{\left|\int_{-\infty }^{\infty }S\left(f\right)H\left(f\right)e^{-j2\pi fT_{b}}df\right|}^{2}}{\frac{N_{0}}{2}\int_{-\infty }^{\infty }{\left|H\left(f\right)\right|}^{2}\;df}$$

To minimize the SNR, Schwartz inequality is employed. It states that for any two conjugate functions $f_{1}\left(t\right)$ and $f_{2}\left(t\right)$:

(11) $${\left|\int_{-\infty }^{\infty }f_{1}\left(t\right)f_{2}\left(t\right)dt\right|}^{2}\leq \;\int_{-\infty }^{\infty }{\left|f_{1}\left(t\right)\right|}^{2}dt\times \int_{-\infty }^{\infty }{\left|f_{2}\left(t\right)\right|}^{2}dt$$

Applying Schwartz inequality to the Equation (10) and considering $f_{1}$ to be H(f) and $f_{2}$ to be $S\left(f\right)e^{-j2\pi fT_{b}}$, the following Equation for the SNR is derived:

(12) $$\frac{S_{p}}{N_{p}}\leq \frac{\int_{-\infty }^{\infty }{\left|H\left(f\right)\right|}^{2}df\times \int_{-\infty }^{\infty }{\left|S\left(f\right)e^{-j2\pi fT_{b}}\right|}^{2}df}{\frac{N_{0}}{2}\int_{-\infty }^{\infty }{\left|H\left(f\right)\right|}^{2}\;df}$$

(13) $$\frac{S_{p}}{N_{p}}\leq \frac{2}{N_{0}}\;\int_{-\infty }^{\infty }{\left|S\left(f\right)\right|}^{2}df$$

where $\int_{-\infty }^{\infty }{\left|S\left(f\right)\right|}^{2}df$ is the energy of the bit signal and can be therefore written as $E_{s}$. Hence, the SNR is expressed as:

(14) $$\frac{S_{p}}{N_{p}}\leq \frac{2}{N_{0}}\;E_{s}$$

According to the Schwartz inequality, this only holds when

(15) $$h\left(t\right)=\;s*\left(T_{b}-t\right)$$

This means that the filter transfer function should have the exact shape and size of the logic ‘1’ bit. In the case of the proposed experiment, this translates to a square-shaped signal of magnitude 4.5 V and duration $T_{b}$. In case of a logic ‘1’ bit being transmitted as s(t), the output of the matched filter is a triangle of base width $2T_{b}$, with a peak at $T_{b}$ of magnitude $E_{s}$. Hence, the maximum output is achieved every $T_{b}$, corresponding to the peak of the triangle.

**Figure 9:**
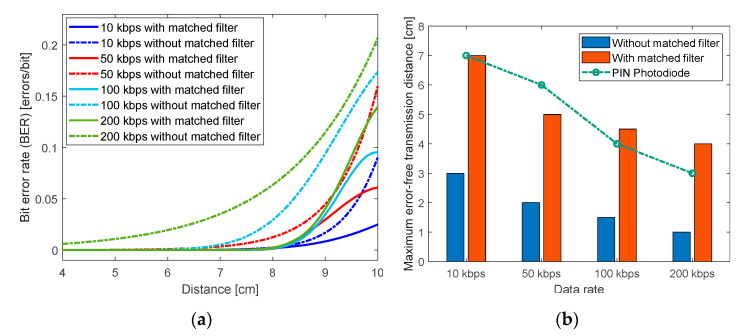
Effect of the matched filter on improving the system performance regarding (**a**) the bit error rate (BER) at different distances and (**b**) the maximum error-free transmission distance.

For the proposed system, a matched filter is designed to improve the BER at every speed and increases the error-free distance as depicted in Figure 9a. A 90% BER improvement is obtained at 200 kbps when the distance is 5 cm. The most significant improvement of the matched filter technique is measured at the 200 kbps, with an improvement averaging 60% for all distances. Figure 9b compares the maximum error-free transmission distances with and without matched filter as well as with those achieved when employing a regular PIN photodiode as photodetector. The bar chart demonstrates that applying the matched filter technique increases the error-free transmission distances for the 10 kbps transmission data rate case from around 3 cm to almost 7 cm. This matches the performance of the LED photodetector to that of a PIN photodiode. At 200 kbps the LED-to-LED link with matched filter exceeds the error-free transmission distance achieved by the LED-to-PIN photodiode link at photovoltaic mode.

The power consumption of the whole system is measured at 110 mW in the worst-case scenario when the transmitter LED is turned on for emitting (which consumes almost 100 mW) and the photodetection circuit is detecting (consuming the remaining 10 mW). The average power consumption is expected to be half of this value assuming equal probabilities of ‘1’ and ‘0’ bits because the transmitter LED is then switched off half of the transmission time. The power consumed by the photodetection circuit can also be slightly reduced by programming an event-driven turning on of the detector circuit: With the help of low-power comparators, the detection circuit is turned on only in response to system interrupts and stays off otherwise [25].

## 4. Conclusions

This paper proves the feasibility of a low-complexity LED-to-LED communication link based entirely on RGB LEDs as transmitters and receivers. The novelty of this research is threefold: First, it proposes a low-complexity design based only on off-the-shelf RGB LEDs to keep the overall cost and power consumption at a minimum to allow for easy integration in IoT devices. Second, it derives a formula for the LED photodetector response at varying distances and empirically proves that the noise of such detector is AWGN. Finally, knowing the noise power and its distribution, it proposes a matched filter technique to increase the system performance even above that using a regular PIN photodiode.

Without matched filter, successful OOK modulated signal detection is possible at data rates up to 200 kbps over a distance up to 3 cm with BER < 10^−3^ and the distance rises to 6 cm at a transmission speed of 10 kbps. Employing the proposed matched filter technique results in a maximum error-free transmission distance comparable to that of a regular PIN photodiode as detector. The results prove that using that technique, the BER improves by up to 90% at a transmission speed of 200 kbps over a 5 cm distance.

Although the achieved data rates are considered low in comparison to other VLC systems, it is sufficient for most small IoT devices that require sending only status flags and small chunks of information, such as toy-to-toy communication. Moreover, the paper proves that the system performance lags due to the much smaller active area of the RGB LED compared to a single-coloured red LED. On the cost of increasing the complexity, the system performance could be further optimized using lenses, concentrators, pre-processing, and further post-processing techniques along with more complex modulation schemes such as OFDM.
